# MAKING IT WORK: PEAK 2.0 PROGRAM EVALUATION RESEARCH

**DOI:** 10.1093/geroni/igz038.2052

**Published:** 2019-11-08

**Authors:** Maggie Syme, Gayle Doll, Laci Cornelison, Migette Kaup

**Affiliations:** Kansas State University, Manhattan, Kansas, United States

## Abstract

Research opportunities from our organizational partnerships allow us to pursue answers to questions about PCC outcomes that have largely been out of reach for the field. Our program evaluation research agenda includes groundbreaking, short-term clinical outcomes from older Kansans living in nursing facilities implementing increasingly levels of PCC, including a 49% lower prevalence of depression for residents in strong PCC adopting homes (Hermer et al., 2017; Hermer et al., 2018). Yet there have been several challenges to achieving these goals that are atypical to the common academic research process. This presentation will further highlight the clinical outcomes association with PEAK 2.0, our continued research partnership with the state, and highlight some of the unique opportunities and challenges large-scale research with a state organization presents.

